# The Interplay between the Gut and Ketogenic Diets in Health and Disease

**DOI:** 10.1002/advs.202504249

**Published:** 2025-08-23

**Authors:** Chunlong Mu, Jong M. Rho, Jane Shearer

**Affiliations:** ^1^ Faculty of Kinesiology & Department of Biochemistry and Molecular Biology Cumming School of Medicine. University of Calgary Calgary AB T2N 1N4 Canada; ^2^ Department of Microbiology Immunology and Infectious Diseases Cumming School of Medicine University of Calgary Calgary AB T2N 1N4 Canada; ^3^ Alberta Children’s Hospital Research Institute University of Calgary Calgary AB T2N 1N4 Canada; ^4^ Department of Pediatrics, Yale School of Medicine Yale University New Haven CT 06510 USA

**Keywords:** gut microbiome, gut physiology, gut barrier, host–microbiome interaction, intestinal Immunity, ketogenic diet, microbiome–gut–brain axis

## Abstract

The gut plays a central role in translating dietary signals into systemic health effects, making it a key mediator of the ketogenic diet (KD), a high fat, low carbohydrate regimen. This review synthesizes current knowledge on the interaction between the KD and the gut, emphasizing gut‐mediated mechanisms as an interface between dietary interventions and systemic health outcomes, spanning gastrointestinal to neurological health. Topics address gut physiology (gut digestion and absorption, epithelial nutrient sensing, gut motility), intestinal immunity (covering innate, adaptive, and antiviral responses), and extracellular to intracellular processes (i.e. mitochondrial function, stem cell fate, and intestinal circadian rhythm). Special focus is given to the gut microbiome, including both bacterial and fungal communities and how the KD modulates them in conditions such as epilepsy, obesity, traumatic brain injury, and multiple sclerosis. Innovative methods for tailoring the KD, including the use of alternative formulations, ketone esters, and microbiome‐focused interventions such as prebiotics and probiotics are examined. Strategies to maximize the diet's benefits while reducing potential side effects are considered. Together, these insights herein offer a comprehensive framework for understanding the interactions between the KD and the gut, a prerequisite for optimizing the overall health benefits of metabolism‐based treatments.

## Graphical abstract

This review synthesizes current knowledge on the interaction between ketogenic diets and the gut, covering gut physiology, intestinal immunity, and the gut microbiome. It explores innovative methods for tailoring the diet to maximize benefits while reducing side effects. Emphasis is placed on gut‐mediated mechanisms as a critical interface between diet and systemic health outcomes, particularly in gastrointestinal and neurological health.

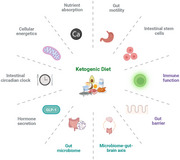

## 1 Introduction

The ketogenic diet (KD) emerged as a medical intervention in the early 1920s and was designed to mimic the metabolic effects of fasting. Since its inception, there has been mounting evidence for the utility of the KD as a metabolic therapy for various medical – and especially, neurological conditions. A classic KD typically contains fats in combination with protein + carbohydrates in the ratio of 3:1–4:1 based on the macronutrient content. The principal objective of the diet is to shift the body's energy source from glycolytic to intermediary metabolism, and notably the production of ketone bodies in the liver through the oxidation of fatty acids. Acetoacetate and the reduced form, β‐hydroxybutyrate (BHB), are the primary ketone bodies utilized by the brain. The brain is a highly energy‐demanding organ, consuming ≈20% of the body's oxygen at rest. Leveraging ketone metabolism to support brain energy demands has opened innovative avenues for treating neurological disorders.^[^ ^1^ ^]^ In the context of epilepsy, ketone bodies also exhibit anticonvulsant effects in animal models.^[^ ^2^, ^3^ ^]^ Beyond ketone production, the KD affects systemic metabolism including fatty acid, branched‐chain amino acid, and tryptophan metabolism.^[^ ^4^ ^]^ Alternative KD formulations, such as medium‐chain triglyceride (MCT) KD, modified Atkins diet, and low‐glycemic‐index treatments, are also widely used.

Recent studies shed light on the utility of the KD against a spectrum of metabolic and neurological disorders. The KD has demonstrated therapeutic potential in autistic spectrum disorder,^[^ ^5^, ^6^ ^]^ glucose transporter 1 deficiency syndrome,^[^ ^7^ ^]^ relapsing multiple sclerosis,^[^ ^8^ ^]^ obesity,^[^ ^9^ ^]^ traumatic brain injury,^[^ ^10^ ^]^ and Alzheimer's disease.^[^ ^11^ ^]^ Preclinical studies further support its protective role in conditions such as drug‐resistant epilepsy,^[^ ^12^ ^]^ infantile spasms,^[^ ^13^, ^14^ ^]^ Alzheimer's disease,^[^ ^15^ ^]^ and high‐fat diet‐induced obesity.^[^ ^16^ ^]^ The putative mechanisms underlying these benefits include, but are not limited to, the regulation of cellular bioenergetics, mitochondrial permeability transition, neurotransmitter production, epigenetic modifications, and reduced inflammation in the central nervous system.^[^ ^17^, ^18^ ^]^ This evidence highlights the significant potential of the KD in ameliorating disease. As such, a comprehensive understanding of how the KD affects the gut is essential to further elucidating its relevant mechanisms of action.

The gut is the first organ encountering the fat‐rich KD. Dietary ingredients, including lipids and carbohydrates, serve as basic nutritional substrates for both the intestinal epithelium and the gut microbiota.^[^ ^19^ ^]^ Given the substantial dietary changes in the KD compared to a regular diet, widespread and complex responses in the gut are expected. These responses encompass key components of gut health, including the gut microbiome, various epithelial cell types, and physiological processes that connect gut function to overall systemic health. The gut harbors the largest external surface area in the body – sensing diverse signals, including those from commensals, pathogens, and injurious chemicals. To precisely maintain the gut and systemic homeostasis, the gut utilizes a series of fine‐tuning systems, such as the enteroendocrine hormone system, intrinsic and extrinsic innervation, barrier function, immune defense,^[^ ^20^ ^]^ and gastrointestinal mechanosensation circuitry.^[^ ^21^ ^]^ These systems collectively mediate the gut's interactions with dietary and luminal signals. In addition to regulating intestinal functions, the gut exerts systemic influences by modulating the activities of remote organs, such as adipose tissue, liver, lung, and brain, via gut microbiota‐derived molecules.^[^ ^22^, ^23^ ^]^ The interorgan dialogue has catalyzed emerging interdisciplinary discoveries, uncovering new gut‐based treatments and mechanisms. Overall, the gut has multifaceted connections to whole body health.

**Figure 1:**
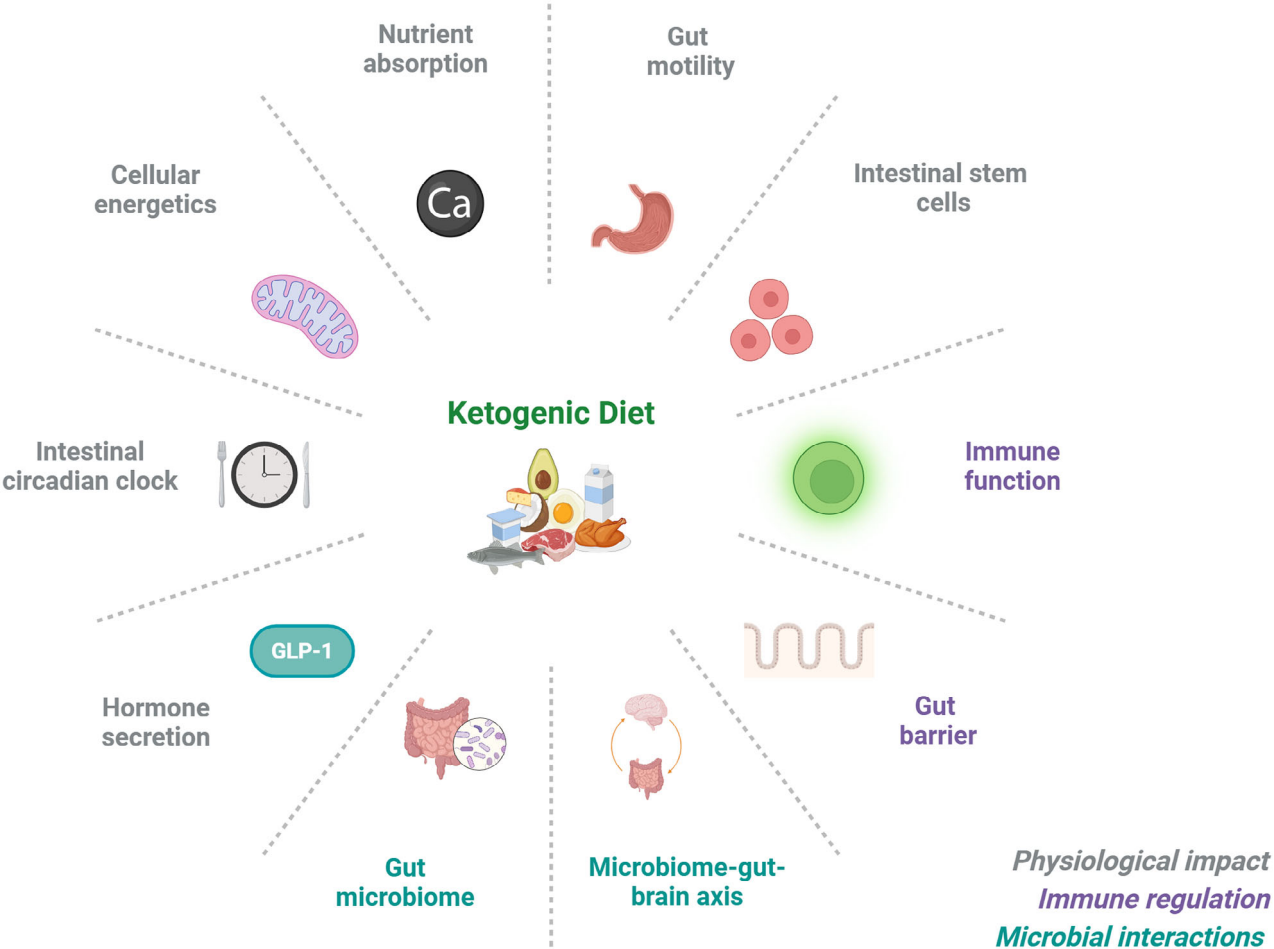
Summary of the effects of the ketogenic diet on the gut. The ketogenic diet affects multiple aspects of gut physiology (gut digestion and absorption, epithelial nutrient sensing, gut motility), intestinal immunity (innate, adaptive, and antiviral responses) as well as extracellular and intracellular processes (i.e. mitochondrial function, stem cell fate, and intestinal circadian rhythm).

Given the importance of the gut as a key interface between dietary intervention and systemic health outcomes, this review aims to provide a comprehensive overview of how the KD affects the gastrointestinal system. We provide a multidimensional perspective that spans gut physiology and intestinal immunity, intracellular and extracellular processes (such as mitochondrial function and stem cell fate) as well as microbial composition and mechanistic insights into the gut microbiome's role in health and disease. This review is structured to follow a logical sequence of interconnected events within the gut, encompassing topics including intestinal lipid processing, motility, barrier integrity, immune responses, circadian rhythms, and the gut microbiome, as illustrated in **Figure** **1**.

## 2 Physiological Regulation and Mechanisms of the Ketogenic Diet

### 2.1 Physiological Regulation of the Gut by the Ketogenic Diet

#### 2.1.1 Nutrient Digestion and Absorption

The gut is the primary organ responsible for nutrient digestion and absorption. The small intestine absorbs more than 95% of dietary fat.^[^ ^24^ ^]^ Dietary lipids undergo tightly regulated processes for triacylglycerol absorption, including luminal hydrolysis (emulsification with bile acids, hydrolysis by pancreatic lipase), uptake by enterocytes (the major absorptive epithelial cells lining the villi), reesterification in the endoplasmic reticulum, triacylglycerol synthesis, and chylomicron formation mediated by apolipoprotein B and microsomal triglyceride transfer protein. A major biochemical outcome of the KD is the elevation of ketone bodies (e.g., BHB and acetoacetate) and free fatty acids with a corresponding decrease in glucose levels.

Lipid processing in the gut significantly influences systemic lipid metabolism. A comprehensive review of gut lipid handling in health and disease can be found here.^[^ ^25^ ^]^ Given the elevated fat content of the KD, efficient function of the small intestine becomes crucial to managing increased lipid flux. At the morphological level, an increase in intestinal villi length has been observed in the small intestine following the KD,^[^ ^26^, ^27^ ^]^ facilitating adaptations to the differential nutrient flux. On a cellular level, an upregulation in lipid processing, involving factors such as microsomal triglyceride transfer protein and CD36, plays a mechanistic role in lipid handling with KD treatment.^[^ ^26^ ^]^ Consequently, systemic lipid profiles may change, as reflected by alterations in blood triglyceride levels. For instance, an increase in plasma triglycerides was found in children with difficult‐to‐control seizures on the KD,^[^ ^28^ ^]^ reflecting the increased dietary lipid content of the diet. It is important to note that dyslipidemia is one common side effect of long‐term KD administration. As such, frequent monitoring of blood lipid levels is necessary to avoid this potentially detrimental impact following KD initiation.

**Figure 2:**
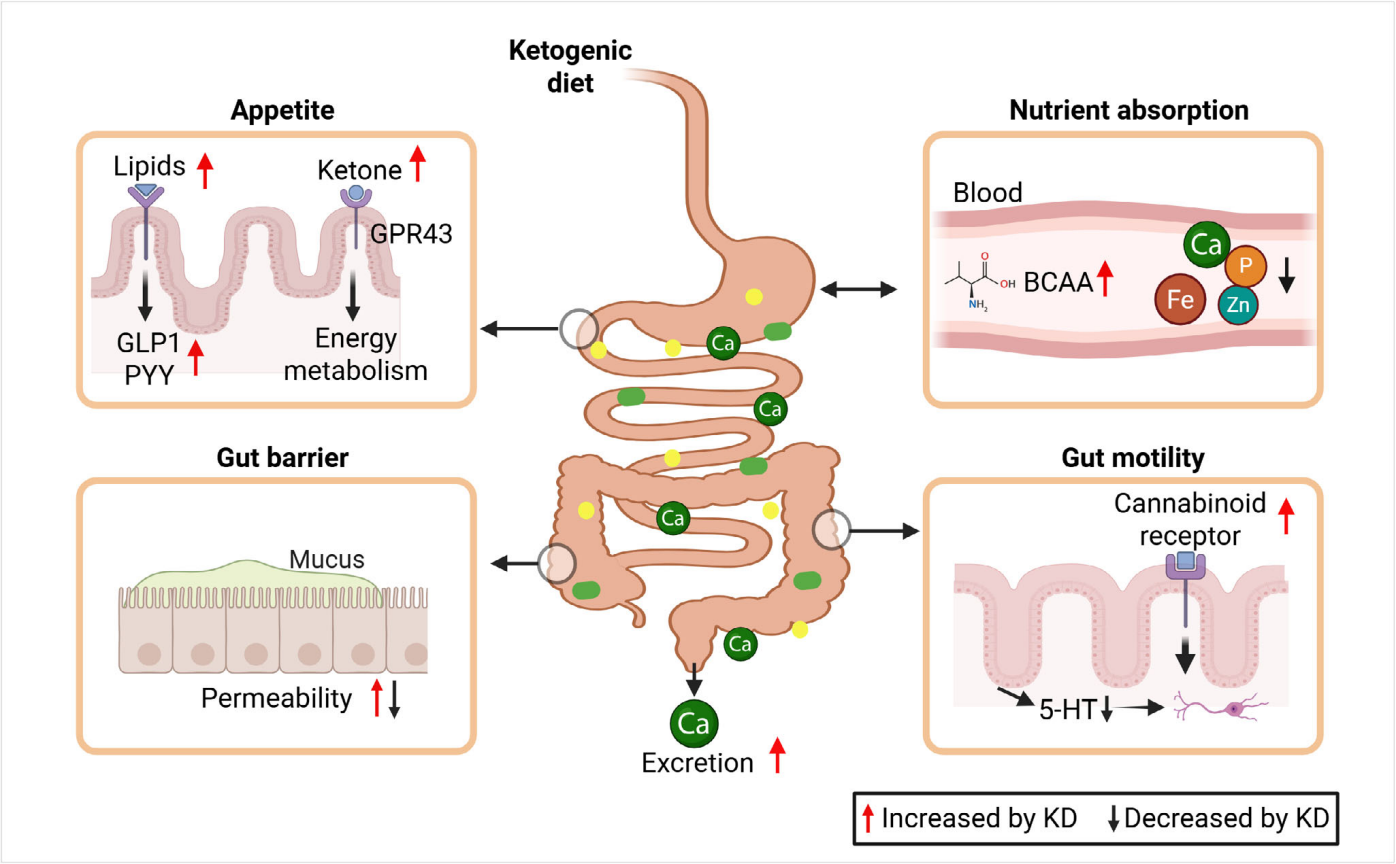
Impact of the ketogenic diet on nutrient absorption and gut motility. The ketogenic diet affects appetite, nutrient absorption, barrier function, and gut motility. Abbreviations: 5‐HT, 5‐hydroxytryptamine (serotonin); BCAA, branched‐chain amino acid; BHB, β‐hydroxybutyrate; GLP1, glucagon‐like peptide‐1; GPR43, G‐protein coupled receptor 43; KD, ketogenic diet; PYY, peptide YY.

Amino acids are not only the building blocks for protein synthesis but also crucial molecules for physiological processes such as ketogenesis and neurotransmitter synthesis. The small intestine, particularly the jejunum, is the main site for the digestion, absorption, and transport of amino acids. Alterations of amino acid profiles in blood^[^ ^4^, ^29^, ^30^ ^]^ and brain^[^ ^31^ ^]^ have been reported with the KD (**Figure** **2**). Branched‐chain amino acids (BCAAs), such as leucine and isoleucine are ketogenic and can be catalyzed to produce acetyl‐CoA, a ketone body precursor that readily enters the brain.

Energy metabolism is fundamental for maintaining cellular homeostasis, especially in the small intestine where there is rapid epithelial cell turnover. Efficient energy supply is a driving force to synthesize antimicrobial molecules and transport nutrients into the intracellular space. Intestinal epithelial cells can utilize various energy sources, but glutamine is a major oxidative fuel followed by glutamate, aspartate, and lactate among others (e.g., glucose).^[^ ^32^, ^33^ ^]^ Following KD, there is an increased reliance of epithelial cells on ketone bodies over their preferred glutamine as energy source. Elevated ketone levels may also interfere with amino acid metabolism, as observed in diabetic ketosis, where increased BHB in the jejunum villi–crypt axis reduces glutaminase activity, impairing glutamine oxidation.^[^ ^34^ ^]^ However, the impact of the KD on intestinal amino acid levels remains unclear. Since the amino acid utilization in the gut affects peripheral availability, the evidence of reduced blood glucogenic amino acids (e.g., glutamine, proline) following the KD^[^ ^4^ ^]^ suggests potential alterations in the gut. In contrast to enterocytes in the small intestine, colonocytes in the large intestine use butyrate as a major energy source. Butyrate comes from the microbial fermentation of complex and simple carbohydrates. The low carbohydrate content in KD often results in reduced carbohydrate fermentation and thus production of short‐chain fatty acids. This hypothesis is supported by a clinical study showing the reduction of fecal butyrate concentration in epilepsy after one month of KD administration.^[^ ^35^ ^]^ These findings raise another intriguing question: how do the colonocytes compensate for the shortage of butyrate while preserving function? One possibility is the potential use of ketone bodies as an alternative energy source.

Minerals such as calcium, zinc, selenium, and iron – essential for normal physiological function – are also affected by the KD (Figure 2). These minerals are particularly important for the growth and development of children, supporting the immune system and bone mineralization. Again, the small intestine is responsible for absorbing and transporting most of these minerals via epithelial cells. Mineral deficiencies encompassing calcium, phosphorous, zinc, and iron have been a perennial concern associated with long‐term KD use.^[^ ^36^, ^37^ ^]^ For instance, mineral deficiencies have been reported in children with medically intractable epilepsy after 15 months on the KD.^[^ ^38^ ^]^ Decreased levels of selenium and magnesium have also been observed in children on a classical KD.^[^ ^39^ ^]^ Deficiencies may result from synergistic effects involving multiple organs. From a digestive perspective, KD reduces calcium digestibility, leading to increased fecal calcium secretion.^[^ ^40^ ^]^ The decreased absorption of calcium could possibly limit its availability in the portal vein and consequently cause lower levels in the liver^[^ ^41^ ^]^ and blood.^[^ ^42^ ^]^ Moreover, reductions in phosphorous, copper, zinc, and selenium have been documented in the liver following the KD.^[^ ^41^ ^]^ These deficiencies may also arise from the consumption of poor mineral‐containing foods, an unbalanced diet,^[^ ^43^ ^]^ or KD‐induced digestive alterations.^[^ ^40^ ^]^ Unfortunately, oral mineral supplementation combined with the KD does not meet the demand in children with refractory epilepsy.^[^ ^37^ ^]^ Clinically, the effects of mineral deficiencies following a KD require further investigation. From a physiological perspective, it is notable that little to no evidence exists regarding changes in mineral metabolism at the interface of the intestinal epithelium. A detailed exploration of this area could provide valuable insights for refining KD treatment strategies or developing gut‐based interventions to mitigate related complications.

#### 2.1.2 Nutrient Chemosensing and Enteroendocrine Hormone Modulation

The gut is widely recognized as an endocrine organ. It produces a battery of hormones to regulate intestinal nutrient absorption, insulin release, and appetite.^[^ ^44^ ^]^ A direct link between nutrients and hormone release is the intestinal chemosensory system lining the intestinal epithelium, which is comprised of diverse taste receptors, mostly in the form of G‐protein‐coupled receptors (GPRs) such as calcium‐sensing receptor, Taste receptor type 1 member 1 and member 3.^[^ ^45^ ^]^ GPRs are the largest family of receptors in cellular membranes that participate many physiological processes. Dietary fats, proteins, carbohydrates, and their derived metabolites are detected by the GPRs to stimulate the production of gastrointestinal hormones, including cholecystokinin, peptide YY (PYY), and glucagon‐like peptides 1 and 2 (GLP‐1, GLP‐2).

The KD is known as a weight loss strategy for obesity management. One of the primary mechanisms by which the KD promotes weight loss is through appetite suppression. For instance, dietary lipids trigger the production of GLP‐1 and PYY by the enteroendocrine cells (Figure 2), promoting satiety effects.^[^ ^45^, ^46^, ^47^ ^]^ In obese men, switching from a basal diet to an isocaloric KD has been shown to elevate levels of fasting ketone bodies, adiponectin, C‐reactive protein, and gastric inhibitory peptide.^[^ ^48^ ^]^ Gastric inhibitory peptide is a major gastrointestinal peptide produced by the enteroendocrine K‐cells in the duodenum and upper jejunum that inhibits gastrointestinal motility. Beyond sensing lipids, ketone bodies from lipid oxidation and ketogenesis can also be sensed by GPRs. BHB is likely recognized by GPR109A and GPR41, while acetoacetate could be recognized by GPR43.^[^ ^49^ ^]^ For instance, acetoacetate induced by KD can activate GPR43 to regulate lipoprotein lipase activity and energy production.^[^ ^50^ ^]^ Such mechanisms provide a compelling scientific rationale for exploring the role of ketone bodies as signaling molecules in the regulation of metabolism.

Ketone esters, including D‐1,3‐butanediol monoester and R,S‐1,3‐butanediol acetoacetate diester, are esters of BHB or acetoacetate. To mimic the ketonemia observed during KD, ketone esters have been developed to allow for better tolerability and rapid release of ketones in epilepsy therapy.^[^ ^51^ ^]^ Of note, ketone esters can recapitulate some effects of the KD on the enteroendocrine system. In individuals of normal weight, consumption of ketone esters increases postprandial plasma insulin, ghrelin, GLP‐1, and PYY levels, while also reducing appetite.^[^ ^52^ ^]^ In summary, the KD's role in enhancing satiety via the enteroendocrine system underscores its function as a critical mediator of gut–brain interactions. Understanding nutrient chemosensing and hormone dynamics in the KD could pave the way for dietary strategies targeting enteroendocrine modulation.

#### 2.1.3 Gut Motility

Gut motility refers to the movement of muscles in the gastrointestinal tract. It is regulated by multiple physiological inputs, including neurotransmitters (e.g., serotonin) that regulate the enteric nervous system, gastrointestinal hormones (e.g., gastrin), and the autonomic nervous system. Dysfunction in gut motility can result in conditions such as diarrhea and constipation. Changes in the composition of dietary macronutrients, such as carbohydrates, fibers, and fats, have varying effects on gut motility.^[^ ^53^, ^54^ ^]^ This introduces both challenges and opportunities to manage gut motility.

Serotonin is a major neurotransmitter regulating gut motility. Serotonin receptor activation regulates the contraction and relaxation of longitudinal and circular muscles, as well as peristaltic and secretory reflexes. Metabolically, 5‐hydroxy indoleacetic acid is the downstream product of serotonin metabolism. The KD has been found to reduce 5‐hydroxy indoleacetic acid in the cerebrospinal fluid of epilepsy patients,^[^ ^55^ ^]^ although the effects on the gut serotonin system remain unclear. A growing body of evidence indicates that serotonin synthesis can be regulated by gut microbes.^[^ ^56^, ^57^ ^]^ This raises the possibility that the KD may alter gut microbes and consequently the serotonin system; this is an intriguing hypothesis and warrants further investigation.

Irritable bowel syndrome (IBS) is a commonly diagnosed gastrointestinal disorder worldwide. It is characterized by symptoms such as abdominal pain, bloating, and altered bowel movements. Experimental evidence has linked IBS with increased serotonin levels and reduced serotonin reuptake in the gut.^[^ ^58^, ^59^ ^]^ In a rat model of IBS induced by early life maternal deprivation, colonic serotonin levels were elevated in animals fed a control diet while the KD reduced colonic serotonin and improved gut function (Figure 2),^[^ ^60^ ^]^ suggesting a beneficial effect on gut motility. The impact of KD on the serotonergic system and gut motility presents a potential therapeutic approach for managing gut function in IBS.

In addition to the serotonergic system, the endocannabinoid system is also involved in regulating gut function. Cannabinoid receptors are transmembrane G‐protein‐coupled receptors and part of the endocannabinoid system. They are mainly expressed in enteric and central neurons to regulate gut motility. Activation of cannabinoid receptors reduce gut discomfort by regulating various physiological processes, including appetite, pain sensation, mood, and inflammation.^[^ ^61^ ^]^ Interestingly, the KD may upregulate the expression of cannabidiol receptors in the colon.^[^ ^62^ ^]^ Following these observations, cannabidiol microemulsions have proven effective in ameliorating IBS–diarrhea by decreasing the fecal water ratio and upregulating the expression of tight junction proteins in the colon.^[^ ^62^ ^]^ Although there is relatively limited evidence, there may be a role of cannabinoid receptors in mediating these changes.

Clinically, gut motility‐related comorbidities are significant challenges affecting adherence to the KD, particularly in patients with epilepsy. Gut motility is governed by a complex interplay of physiological processes, spanning cellular to neurochemical signaling mechanisms. The KD has the potential to influence this intricate system through multiple pathways. However, our understanding of how the KD regulates gut motility remains limited. In essence, understanding how KD regulates gut motility is crucial for developing alternative treatments to effectively manage gut‐related complications.

### 2.2 Intracellular Mechanisms Influenced by the Ketogenic Diet

#### 2.2.1 Mitochondrial Metabolism

Mitochondria are commonly referred to as the powerhouses of the cell because they play a crucial role in producing energy required for various cellular functions. It is well established that the restoration of brain mitochondrial function is an important mechanism mediating the protective effects of the KD against neurological disorders such as epilepsy.^[^ ^18^ ^]^ Nevertheless, the role of the KD on intestinal mitochondrial function remains poorly understood. Mitochondria are essential to maintain intestinal epithelial homeostasis.^[^ ^63^ ^]^ It is important to note that gut mitochondrial dysfunction is implicated in inflammatory bowel disease (IBD),^[^ ^64^ ^]^ colitis,^[^ ^65^ ^]^ and colorectal cancer.^[^ ^66^ ^]^

**Figure 3:**
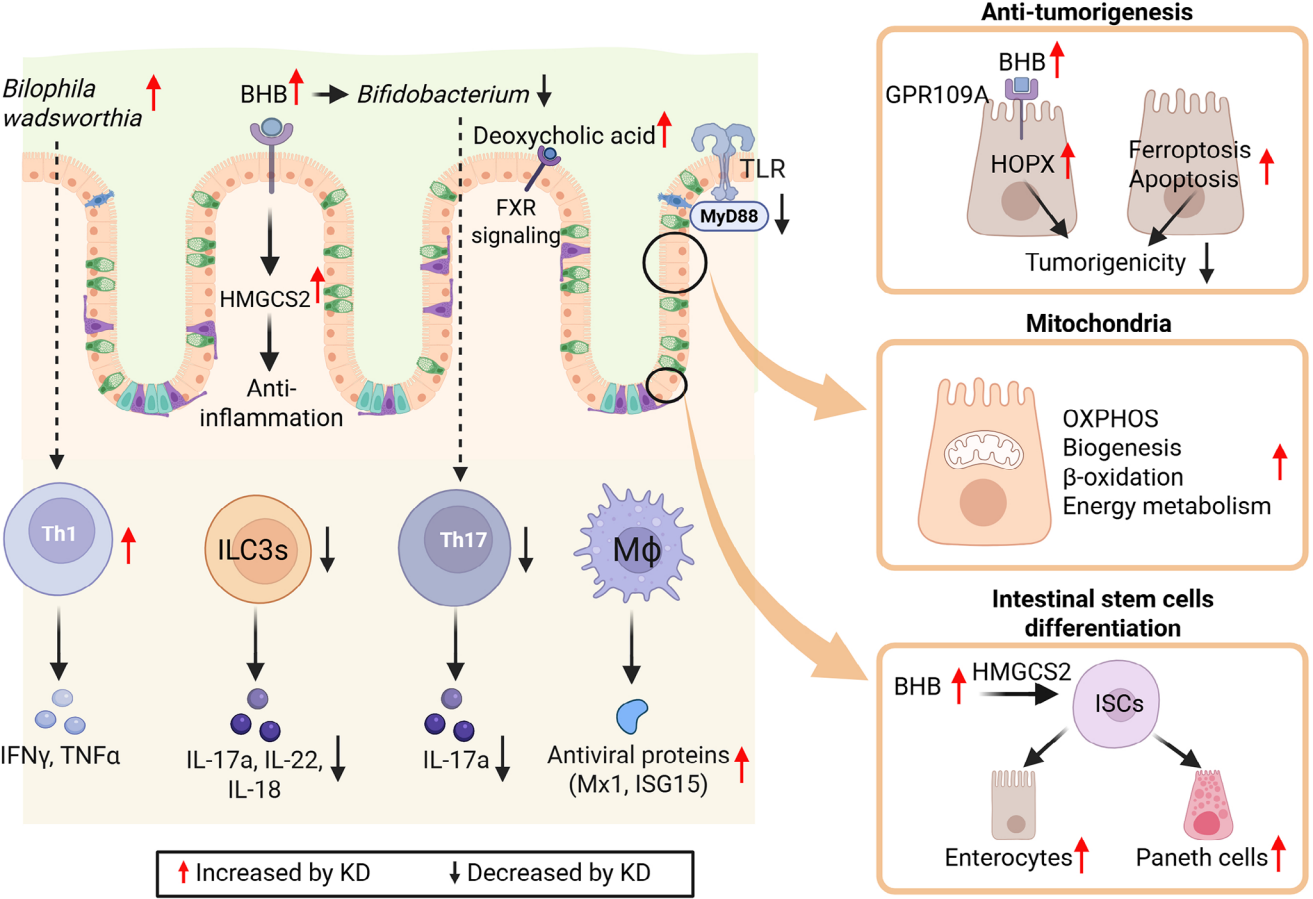
Impact of the ketogenic diet on cellular process and intestinal immunity. The ketogenic diet affects tumorigenesis, mitochondria function, and intestinal stem cells via a variety of pathways. Abbreviations: BHB, β‐hydroxybutyrate; FXR, farnesoid X receptor; GPR109A, G‐protein coupled receptor 109A; *HMGCS2*, 3‐hydroxy‐3‐methylglutaryl‐coa synthase 2; *HOPX*, homeodomain‐only protein; IFNγ, interferon‐gamma; IL‐17a, interleukin 17a; IL‐18, interleukin 18; IL‐22, interleukin 22; ILC3, group 3 innate lymphoid cells; ISCs, intestinal stem cells; ISG15, interferon‐stimulated gene 15; KD, ketogenic diet; MΦ, macrophages; Mx1, MX dynamin like GTPase 1; MyD88, myeloid differentiation primary response 88; OXPHOS, oxidative phosphorylation; Th1, T helper 1 cells; Th17, T helper 17 cells; TLR4, toll‐like receptor 4; TNFα, tumor necrosis factor alpha.

As discussed above, the KD may be of value in ameliorating IBS. There is evidence showing that KD‐mediated protection is related to improvements in mitochondrial function. In a mouse model of IBS, the KD improved inflammation, oxidative stress, mitochondrial biogenesis, and autophagy in the colon^[^ ^67^ ^]^ (**Figure** **3**). Importantly, in the small intestine, the KD upregulates the expression of enzymes related to lipid oxidation and oxidative phosphorylation, indicating the enhancement of mitochondrial function.^[^ ^68^ ^]^ The KD is well‐known to increase circulating BHB levels, and BHB in turn is a broad regulator of mitochondrial function^[^ ^69^ ^]^ via numerous post‐translational mechanisms. For example, BHB levels lower mitochondrial protein acetylation by depleting the acetyl‐CoA pool, thereby boosting the activity of enzymes such as citrate synthase.^[^ ^70^ ^]^ This decrease is linked to enhanced mitochondrial function and greater resistance to inflammatory stressors. Collectively, the published evidence strongly supports the view that the beneficial effects of the KD on the gastrointestinal system may be mediated in part through enhanced intestinal mitochondrial function.

#### 2.2.2 Ketogenic Diet and Intestinal Stem Cell Regulation

Intestinal stem cells (ISCs) are fundamental to maintaining epithelial homeostasis. Fine‐tuned proliferation of ISCs along the crypt–villus axis are essential for gastrointestinal architecture and function, including nutrient absorption and antimicrobial defense.^[^ ^71^ ^]^ ISC homeostasis is regulated by Wnt, Notch, epidermal growth factor, and bone morphogenetic protein signaling amoung others.^[^ ^72^ ^]^

Various dietary patterns, including the KD, a high‐fat diet, and Western diet, can influence stem cell fate.^[^ ^73^, ^74^ ^]^ For instance, the KD can upregulate the expression of 3‐hydroxy‐3‐methylglutaryl‐CoA synthase 2 (*HMGCS2*) along the crypt–villus axis of the small intestine, enhance Notch activity and self‐renewal of intestinal stem cells, Notch intracellular domain nuclear localization, and increase the number and proliferation of ISCs.^[^ ^69^ ^]^ These effects are mediated by BHB via *HMGCS2*‐dependent regulation and its histone deacetylase inhibitor activity^[^ ^69^ ^]^ (Figure 3). This finding is also supported by a study showing that the KD promotes the differentiation of stem cells toward enterocytes and Paneth cells via BHB in a *HMGCS2*‐dependent manner.^[^ ^75^ ^]^ A positive impact of BHB on intestinal stem cells has also been observed in *Drosophila* models. Feeding BHB to *Drosophila* helps maintain midgut stem cell homeostasis during aging by enhancing antioxidative functions, reducing hyperproliferation and DNA damage, and inhibiting heterochromatin instability.^[^ ^76^ ^]^ Based on these observations, it is becoming increasingly clear that the regulation of intestinal stem cell fate can be modified by ketogenesis.

#### 2.2.3 Circadian Rhythms in the Intestinal Epithelium

Circadian rhythms are 24 h cycles that drive daily fluctuations in gene expression at the molecular level. These rhythms play a vital role in maintaining circadian homeostasis that regulate the sleep–wake cycle, hormone secretion, temperature balance, and various metabolic processes.^[^ ^77^, ^78^ ^]^ Recent studies indicate that the KD may influence circadian rhythm behaviors and modify circadian gene expression in peripheral tissues, such as liver, heart, and brain.^[^ ^79^ ^]^ Notably, the KD may also affect the intestinal circadian clock. In mice, the KD altered levels of BHB in a circadian‐dependent manner with the lowest response at Zeitgeber Time (ZT) 8 (8 h into the light phase) and peaking at ZT20 in the ileum tissue, a change that corresponds to cyclic histone deacetylase activity causing acetylation of peroxisome proliferator‐activated receptor‐responsive element of *HMGCS2*, acyl‐CoA thioesterase 2, and the carnitine palmitoyltransferase 1A promoter.^[^ ^68^ ^]^ Similar phenomena have also been observed in serum but not in liver.^[^ ^68^ ^]^ Since gut homeostasis is tightly affected by the circadian clock and gut microbiota,^[^ ^80^, ^81^ ^]^ this evidence raises a novel mechanism linking intestinal circadian rhythms to the health impacts of the KD.

Thus far, there are limited studies on the effects of KD on the circadian rhythm. In a mouse study, 2 weeks of MCT‐based KD caused changes in body physiology, such as the reduction of rapid‐eye‐movement sleep and the slight reduction in circadian rhythms of wheel‐running activity, but minimal to no effect on circadian gene expression in the hypothalamus, heart, liver, and skeletal muscle.^[^ ^82^ ^]^ It is of interest to further investigate how the KD affects intestinal circadian rhythm in humans.

## 3 Immune Modulation by the Ketogenic Diet

### 3.1 Intestinal Barrier Function and Ketogenic Diet

The intestinal barrier serves as a physical interface protecting the health of both the intestinal and peripheral systems. The barrier is composed of tightly interconnected layers, extending from the intestinal lumen to the mucosa, and includes key components such as intestinal alkaline phosphatase, mucus, tight junction proteins, antimicrobial proteins, and immunoglobulins.^[^ ^83^, ^84^ ^]^ Tight junction proteins, including claudins, occludin, zonula occludens (ZO), are essential to maintaining the integrity of the intestinal epithelium.^[^ ^85^, ^86^ ^]^ Dysregulation of these proteins are closely linked to disruptions in mucosal homeostasis.^[^ ^87^ ^]^ At present, the effects of KD on the intestinal barrier remain inconsistent and controversial.

#### 3.1.1 Impact in Healthy Subjects

Adverse effects of the KD on intestinal barrier function have been reported. For instance, in wild‐type specific‐pathogen free (SPF) mice, the KD reduced the number of ZO‐1‐positive cells in the ileum and lowered the gene expression of tight junction proteins, including occludin and mucin‐2, together with an increase in blood lipopolysaccharide (LPS)‐binding protein.^[^ ^88^ ^]^ In obese adults, 8 weeks of a very‐low‐calorie KD increases small intestinal permeability, characterized by an increasing lactulose–mannitol ratio and high serum LPS concentration, although significant body weight loss was achieved following KD treatment,^[^ ^89^ ^]^ indicating the intestinal barrier function may be compromised following the diet. Similar conclusions were reached in another human study showing that KD may damage intestinal barrier function by increasing blood LPS‐binding protein and soluble CD14 after endurance exercise consisting of a 25 km race walk.^[^ ^90^ ^]^ LPS is a trigger of proinflammatory response. This evidence highlights the importance of considering intestinal barrier regulation when adopting the KD in healthy individuals.

#### 3.1.2 Impact in Intestinal Diseases

IBD and IBS are major threats to gastrointestinal health. Conditions like IBS and colitis are characterized by compromised gut barrier and immune function.^[^ ^91^, ^92^ ^]^ The impact of the KD on these chronic intestinal diseases is not consistently reported.

The KD has shown promise in alleviating IBS and colitis. In the former, the KD restored IBS‐induced reductions in crypt length, occludin, glucose transporter protein type 1, and cannabinoid receptor 1 in the distal small intestine, all of which may contribute to its beneficial effects in IBS.^[^ ^93^ ^]^ Similar protective effects are also observed in colitis. In a mouse model of colitis, the KD enhanced colonic barrier function by upregulating occludin, ZO‐1, Mucin‐2, and mucin‐producing cells, which contributed to enhanced gut barrier function and lowered serum LPS levels.^[^ ^94^ ^]^ Additionally, recent studies have uncovered a protective effect of the KD against colitis in mice, but the effects on inflammation depend on KD lipid composition (long‐ vs medium‐chain triglycerides)^[^ ^95^ ^]^ or the lipid source (saturated fatty acids vs polyunsaturated linoleic acid).^[^ ^96^ ^]^ These examples provide a foundation for exploring the advantages of a tailored KD in cases of IBS and colitis.

In contrast, another study reported that four weeks of KD worsened dextran sodium sulfate (DSS)‐induced colitis, upregulated proinflammatory cytokines, increased epithelial permeability, and compromised colonic barrier function.^[^ ^97^ ^]^ Although more evidence is needed, these discrepancies likely stem from differences in disease pathogenesis, KD formulations, and physiological factors. Given the substantial burden that IBD and IBS impose on patients, it is important to thoroughly assess the impact of the KD on the intestinal barrier to determine its efficacy and safety in different contexts.

### 3.2 Immune Modulation by the Ketogenic Diet in Health and Diseases

#### 3.2.1 Innate Immunity

The innate immune system is the first line of defense against pathogens. Key components of innate immunity include lymphoid cells and pattern recognition receptor‐medicated signaling. The KD exhibits broad effects on the innate immune system (Figure 3). Innate lymphoid cells (ILCs) are a subset of lymphocytes that lack antigen‐specific receptors and participate in the early immune response against infection. For example, group 3 innate lymphoid cells (ILC3s) have multifaceted roles in either proinflammatory or anti‐inflammatory responses. In intestinal disease, ILC3s can drive the proinflammatory response.^[^ ^98^ ^]^ In a mouse model of colitis, KD alters the gut microbiota by increasing *Akkermansia* and decreasing *Escherichia*/*Shigella*, which leads to the reduction of ILC3s in the colon and ameliorates mucosal inflammation.^[^ ^94^ ^]^ While it is well established that different cell lineages serve distinct functions, the immunological significance of specific cell populations in response to the KD remains underexplored, limiting our understanding of its mechanisms.

Pattern recognition receptors, including Toll‐like receptors 2 and 4, are key immunological sensors detecting signals from microbes to trigger inflammatory responses. These receptors activate downstream signaling pathways, often through myeloid differentiation primary response 88 (MyD88)‐dependent or independent mechanisms.^[^ ^99^ ^]^ Activation of MyD88 typically induces proinflammatory responses. Notably, KD has been shown to downregulate MyD88 expression in the jejunal mucosa, Peyer's patches, and mesenteric lymph nodes of healthy mice,^[^ ^27^ ^]^ suggesting a protective role of KD in modulating immune responses. Overall, the KD's regulation of the innate immune system appears to exert favorable effects on gut health.

#### 3.2.2 Adaptive Immunity

The adaptive immune system is a primary component of immunity, characterized by immunological specificity and memory that involve T and B lymphocytes. T cell subsets, such as T helper 1 cells (Th1) and T helper 17 cells (Th17), contribute to targeted immune responses. Additionally, B cells produce immunoglobulin A (IgA), a class of antibodies that play a significant role in mucosal immunity. Alterations of peripheral adaptive immune following the KD have been reported in human trials.^[^ ^100^, ^101^, ^102^ ^]^

Maintaining homeostatic adaptive immunity involves complex host–microbiome interactions. The impact of the gut microbiome on immune function has attracted considerable attention due to its health implications. The KD can regulate the interaction between the gut microbiome and immune responses. In a mouse model, KD‐induced increases in BHB inhibited the growth of certain *Bifidobacterium* and *Lactobacillus* species that ultimately reduced the population of Th17 cells in the small intestine.^[^ ^103^ ^]^ Conversely, in mice with hypoxia‐induced cognitive impairment, the KD worsened cognitive behavior by enriching *Bilophila wadsworthia*, a bacterium that stimulates intestinal interferon‐gamma‐producing Th1 cells to exacerbate proinflammatory responses.^[^ ^104^ ^]^ Given these data, it appears that the KD can induce context‐specific changes in the gut microbiome and the host immune response depending on health or disease status.

In addition to microbiome‐dependent regulation of the immune response, microbiome‐independent pathways, such as cellular ketogenesis also contribute to KD‐induced effects. *HMGCS2* is the key rate‐limiting enzyme for ketogenesis. Lower *HMGCS2* expression is linked to gut inflammation in IBD.^[^ ^105^ ^]^ However, this effect could be reversed by BHB via a *HMGCS2*‐dependent pathway, reducing proinflammatory chemokine responses in intestinal cells.^[^ ^105^ ^]^ This evidence indicates the immunometabolic role of ketogenesis in regulating gut immunity.

Although the effect of the KD on intestinal immunity in humans is poorly understood, available evidence suggests notable impacts on mucosal immunity. For instance, KD has been shown to increase the concentration of secretory salivary IgA in endurance athletes,^[^ ^106^ ^]^ indicating enhanced mucosal immune activity. Systemic regulation of inflammation has also been observed, as evidenced by reduced serum levels of the inflammatory cytokine interleukin (IL)‐1β with weight loss in obese females following KD.^[^ ^107^ ^]^ Both IgA and IL‐1β exhibit broad immunological effects. Their response to the KD suggests investigation into the links between the gut and systemic adaptive immunity is warranted.

#### 3.2.3 Antiviral Immunity

The antiviral immune system plays a critical role in safeguarding the body against viral infections by orchestrating a complex network of cellular and molecular defenses. This system encompasses both the innate and adaptive immune responses, which function in a coordinated manner to detect, neutralize, and eliminate invading viruses. Type I interferon and interferon‐stimulated gene (ISG) are key players. Interferon activates a signal transduction cascade to stimulate the induction of a wide array of ISGs with antiviral activities.^[^ ^108^, ^109^ ^]^ For example, MX Dynamin Like GTPase 1 (Mx1) is an interferon‐induced dynamin‐like GTPase that antagonizes the replication of several RNA and DNA viruses.^[^ ^109^ ^]^ Similarly, ISG15 is an interferon‐induced 15 kDa ubiquitin‐like protein that regulates the viral replication by conjugation with viral proteins.^[^ ^110^ ^]^ An enhanced expression of ISGs provides antiviral immunity against virus infection. Available evidence supports the role of the KD in regulating antiviral immunity. For instance, in a mouse model of colitis, the KD upregulates the transcriptional expression of proteins in antiviral signaling, such as transcription factors (signal transducer and activator of transcription 1, interferon regulatory factor 7), and antiviral proteins (Mx1, ISG15, 2′‐5′‐oligoadenylate synthetase 2, and interferon‐induced 56 kDa protein),^[^ ^94^ ^]^ which may collectively aid the anti‐inflammatory response in the colon. Interestingly, the KD and ketogenesis appear to be protective against SARS‐CoV‐2 infection in lung by restoring CD4^+^ T cell metabolism and function.^[^ ^111^ ^]^ Meanwhile, the KD also upregulates the expression of ISGs, such as Mx1 and ISG15, in the bronchoalveolar lavage fluid of patients with SARS‐CoV‐2 infection.^[^ ^111^ ^]^ Clinical trials further support the application of the KD to reduce SARS‐CoV‐2 burden in patients with type 2 diabetes mellitus and obesity^[^ ^112^ ^]^ by reducing cytokine storms in these patients.^[^ ^113^ ^]^ These observations highlight the potential of KD as a dietary therapy for enhancing antiviral immunity.

KD‐induced alterations in the gut microbiome may also play a role in its antiviral actions. In a mouse model of herpes simplex virus infection, the KD is protective against herpes simplex virus type 1‐induced neurodegenerative symptoms and neuroinflammation. The role of the gut microbiota was shown to be essential to this response because microbiota depletion by antibiotics weakened KD‐induced protection against the virus.^[^ ^114^ ^]^ Although the underlying mechanisms require further investigation, this observation highlights the possible contribution of gut microbes to antiviral immunity.^[^ ^115^, ^116^ ^]^

#### 3.2.4 Immuno‐Oncology: Ketogenic Diet in Gastrointestinal Cancers

Gastrointestinal cancers account for approximately 26% of global cancer incidence and 35% of all cancer‐related deaths. They involve abnormalities in the intestinal immunity. This section will discuss different types of gastrointestinal cancers in response to the KD.

The KD has been shown to lessen the burden of gastric cancer in preclinical models. In a mouse model of gastric cancer, a KD supplemented with omega‐3 fatty acids and medium‐chain triglycerides effectively reduced the burden of gastric cancer and increased survival.^[^ ^117^, ^118^ ^]^ Consistent with this observation, KD alone or in combination with *Oldenlandia diffusa* extract and curcumin improved the gastric cancer progression, as characterized by the decrease of inflammation, oxidative stress, angiogenesis, as well as an upregulation of miR‐340 in the gastric tissue,^[^ ^119^ ^]^ an epigenetic factor in regulating cellular proliferation and apoptosis. In colorectal cancer, existing evidence supports the beneficial impact of the KD in controlling cancer progression. The KD effectively reduced tumor proliferation in crypt cells in the colon via BHB in a mouse model^[^ ^120^ ^]^ (Figure 3). Mechanistically, BHB could bind to GPR109A leading to the activation of homeodomain‐only protein *HOPX* that inhibits tumorigenicity.^[^ ^120^ ^]^ The reduction in colorectal tumor severity has also been reported in mice bearing subcutaneous C26 colorectal tumors after KD. Finally, the KD exerts a distinct mechanism by promoting tumor ferroptosis,^[^ ^121^ ^]^ an iron‐dependent form of programmed cell death.

The pancreas is another organ in the gastrointestinal system that plays a role in blood glucose regulation and nutrient digestion. Pancreatic cancer is the seventh leading cause of cancer mortality globally.^[^ ^122^ ^]^ In preclinical studies, KD has been shown to enhance the efficacy of the p110α‐specific inhibitor BYL‐719, reducing tumor burden in mice with KPC K8484 pancreatic tumors.^[^ ^123^ ^]^ Moreover, the KD may have synergistic impacts with chemotherapy, including nab‐paclitaxel, gemcitabine, and cisplatin, to suppress tumor growth in mouse models of pancreatic cancer.^[^ ^124^ ^]^ These preclinical results provide a foundation for future clinical investigation.

## 4 Gut Microbiome Modulation by the Ketogenic Diet in Health and Disease

### 4.1 Biogeographical Shifts in the Gut Microbiome Influenced by the Ketogenic Diet

**Figure 4:**
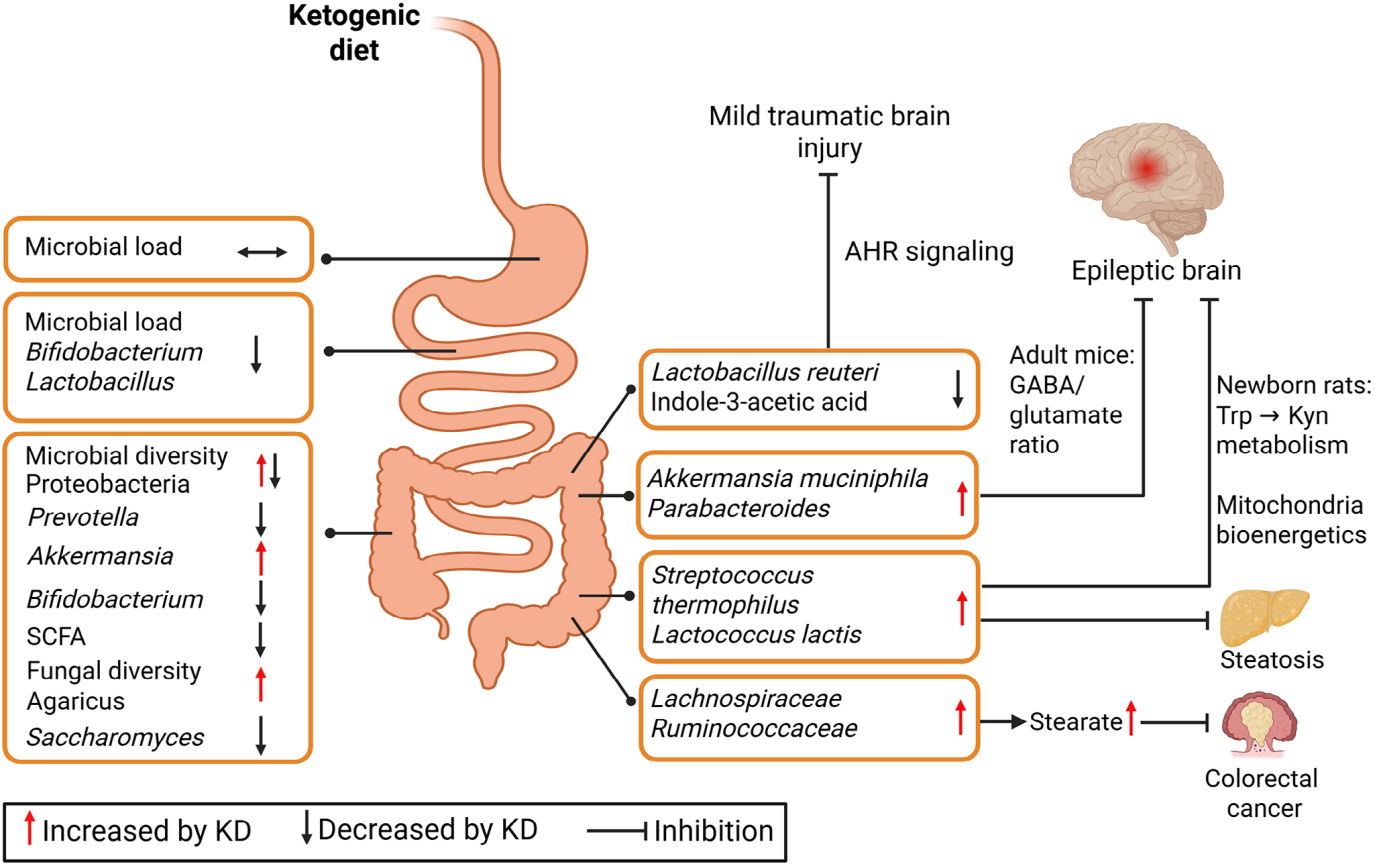
Summary of the effects of the ketogenic diet on the gut microbiome. The ketogenic diet exerts gut location‐dependent impacts. Some of the microbial alterations are mechanistically linked with diseases, such as epilepsy and mild traumatic brain injury. Abbreviations: AHR, aryl hydrocarbon receptor; GABA, gamma‐aminobutyric acid; KD, ketogenic diet; Kyn, kynurenine; SCFA, short‐chain fatty acid; Trp, tryptophan.

Biogeography is the study of species composition within ecosystems, emphasizing the spatial or niche‐specific distribution of species. In the context of the gut microbiome, microbial biogeography focuses on the distinct communities along the gastrointestinal tract, from the stomach to the small and large intestines. The effects of a KD on gut microbiota vary by gut locations (**Figure** **4**).

The stomach contains specific microbes that have adapted to survive in its acidic environment. The impact of the KD on the gut microbiome in the stomach is less studied. Only one study demonstrated that KD does not affect the microbial load in the stomach digesta of mice.^[^ ^125^ ^]^ In the gut, mice fed with KD for 10 days exhibited a reduction in microbial load in both the mucosa and lumen of the small and large intestines.^[^ ^125^ ^]^ Moving along the gastrointestinal tract, the large intestine is the major site for fermentation with a denser microbiome than the stomach and small intestine. In healthy mice, the KD has been shown to enrich *Akkermansia* and *Enterococcus* while reducing *Turicibacter* and *Marvinbryantia* in the cecum and colon.^[^ ^125^ ^]^ An increase in the *Akkermansia* in the colon was also reported in mouse model of colitis after the KD.^[^ ^94^ ^]^ Another study demonstrated that KD inhibits the colonization of *Bifidobacterium* and reduces proinflammatory Th17 cells in the small intestine but not in the colon.^[^ ^103^ ^]^ However, direct comparisons between these studies should be made cautiously due to differences in diet composition, treatment duration, and experimental conditions. Notably, the KD exerts distinct biogeographical effects on the gut microbiome, implicating the importance of considering specific gut locations when addressing research questions related to microbial distribution and function.

### 4.2 Changes in Microbial Composition and Functionality

#### 4.2.1 Nondiseased Conditions

Diet is known as a driver in shaping the gut microbiome. Alterations in the gut microbiome after KD intake have been observed in healthy adult humans,^[^ ^126^ ^]^ rats,^[^ ^127^ ^]^ and mice,^[^ ^128^ ^]^ although different effects are observed. For example, the KD increases *Akkermansia* and reduces *Bifidobacterium* in healthy adults,^[^ ^126^ ^]^ while increasing *Enterococcus*, *Rothia*, and reducing *Lactobacillus* and *Lactococcus* in healthy rats.^[^ ^127^ ^]^ These differences likely reflect variations in species physiology and dietary treatment protocols.

While rodents are valuable research models for exploring the KD's impact on the microbiome, it is important to note that animal genetics and housing environments significantly influence microbiome composition. Notably, KD consistently alters the gut microbiome in healthy mice with different genetic backgrounds by increasing taxa such as *Faecalibaculum*, *Blautia*, *Lactococcus*, and *Akkermansia*, while decreasing *Bifidobacterium* and *Ruminococcaceae* UCG‐005.^[^ ^129^ ^]^ These findings highlight the KD's dominant effect over genetic factors in shaping the gut microbiome, aligning with earlier observations that diet plays a more critical role than genetics.^[^ ^130^ ^]^ Housing conditions also matter; for example, mice housed in conventional environments show a more pronounced microbiome response to KD compared to those in SPF conditions.^[^ ^131^ ^]^ These effects on the fecal microbiome unveil the importance of considering experimental covariables in KD studies.

In addition to microbial composition, microbial metabolism is also affected by the KD. Given the low carbohydrate content of the KD, it is expected to impact the gut microbiome by reducing carbohydrate availability for microbial fermentation. In mice, the KD reduces *Blautia*, *Turicibacter*, and *Enterococcus*, while enriching *Intestinimonas* and *Ruminococcaceae*,^[^ ^88^ ^]^ and reduces short‐chain fatty acid (SCFA) concentrations in the cecum while increasing secondary bile acids such as deoxycholic acid.^[^ ^88^ ^]^ Bile acids regulate the digestion and absorption of fat in the small intestine. However, the overproduction of deoxycholic acid may have detrimental impacts on the large intestine. These alterations have the potential to influence diverse cellular processes, including energy production and inflammation that likely impact epithelial homeostasis.

Beyond its impacts within the gastrointestinal tract, the KD further regulates extraintestinal functions (e.g., the brain) associated with the alteration of the gut microbiome. The gut microbiome's impact on brain function has garnered particular attention due to the well‐documented gut–brain axis.^[^ ^23^ ^]^ Alterations in the gut microbiome are associated with changes in neurochemical function, such as brain connectivity in humans,^[^ ^132^ ^]^ hypothalamic neurotransmitter synthesis in pigs,^[^ ^133^ ^]^ and neurovascular function in mice.^[^ ^134^ ^]^ For example, in SPF mice fed KD, the increased *Akkermansia*, *Lactobacillus*, and *Adlercreutzia*, along with reduced *Turicibacter*, *Dorea*, and *Desulfovibrio*, correlated with improved neurovascular function. These improvements included enhanced blood–brain barrier integrity and reduced amyloid‐beta production,^[^ ^134^ ^]^ suggesting a microbiome‐mediated interaction affecting neural health. While these findings highlight intriguing correlations, more work elucidating the mechanistic connection between the gut microbiome and neurological function is needed.

#### 4.2.2 Microbial Features Associated with Diseases in Clinical Response to KD

The gut microbiome is now widely acknowledged for its influence on disease development through host–microbiome interactions and its potential as a biomarker for disease diagnosis. Microbial alterations induced by the KD have been observed across various conditions, including epilepsy, autism spectrum disorder, Alzheimer's disease, obesity, cancer, and multiple sclerosis.^[^ ^135^ ^]^ The effects of the KD can vary across different diseases, the host's physiology, differences in KD formulations, duration of treatment, and characteristics of study subjects.

Certain microbial alterations following KD appear to be consistent. For instance, increases in *Bacteroidota* taxa, such as *Bacteroides* and *Parabacteroides* (**Table** **1**), have been observed in epilepsy,^[^ ^12^, ^136^, ^137^, ^138^, ^139^, ^140^ ^]^ hypoglycemia,^[^ ^141^ ^]^ and polycystic ovarian syndrome.^[^ ^142^ ^]^ Reductions in *Bifidobacterium* have also been noted in epilepsy,^[^ ^140^, ^143^ ^]^ autism spectrum disorders,^[^ ^144^ ^]^ cognitive impairment,^[^ ^145^ ^]^ and obesity.^[^ ^103^ ^]^ These consistent microbial shifts may serve as potential biomarkers for KD responsiveness.

**Table 1:** Disease conditions with modulated gut microbiota by ketogenic diet.

Disease	Model	Experimental design	Mechanism	References
Epilepsy: drug‐resistant epilepsy	Human (children)	3 months classical KD	Responders with >50% reduction in seizures have higher *L. lactis* subsp. *lactis* and *Bifidobacterium longum* subsp. *longum* and lower *Alistipes shahii*, *Eubacterium rectale*, and *A. muciniphila*	Dahlin et al., 2022^[^ ^55^ ^]^
Epilepsy: drug‐resistant epilepsy	Human (children)	1 weeks KD	Increased *Bacteroides*, decreased *Proteobacteria* and *Cronobacter* in feces	Xie et al., 2017^[^ ^136^ ^]^
Epilepsy: drug‐resistant epilepsy	Human (children)	6 months KD (4:1 ketogenic ratio), post‐KD versus baseline	Increased *Bacteroides*, decreased *Ruminococcaceae*, *Faecalibacterium*, *Actinobacteria*, *Coprobacter*, and *Leucobacter*	Zhang et al., 2018^[^ ^137^ ^]^
Epilepsy: drug‐resistant epilepsy	Human (children)	3 months KD (3–4:1), post‐KD versus baseline	Increased *Escherichia*, decreased *Bifidobacterium*, *E. rectale*, *Dialister*, and microbial carbohydrate metabolism	Lindefeldt et al., 2019^[^ ^143^ ^]^
Epilepsy: drug‐resistant epilepsy	Human (children)	6 months KD	Increased *Subdoligranulum*, *Dialister*, *Alloprevotella*, acetate, decreased *Bifidobacterium*, *Akkermansia*, *Enterococcaceae*, and *Actinomyces*. Patient with good outcome has higher butyrate in feces.	Gong et al., 2021^[^ ^140^ ^]^
Epilepsy: drug‐resistant epilepsy	Human (children)	KD for 12–90 months (average 53.5 months)	A trend toward increased alpha diversity, a trend toward a higher relative abundance of *Bacteroidaceae*, *Ruminococcaceae*, and *Prevotellaceae* species	Freibauer et al., 2025^[^ ^204^ ^]^
Epilepsy: drug‐resistant epilepsy	Human (2–46 yrs)	1 months KD (4:1)	Reduced acetate, propionate, butyrate, and isobutyrate in the feces; lower fecal water genotoxicity, no alteration in fecal water cytotoxicity	Ferraris et al., 2021^[^ ^35^ ^]^
Epilepsy: mitochondrial epilepsy	Human (children)	3 months KD (2:1), KD versus control	Reduced a‐diversity, *Firmicutes*, and *Phascolarctobacterium*, increased *Bacteroides*, *Bacteroides fragilis*	Wang et al., 2023^[^ ^138^ ^]^
Seizures in glucose transporter type 1 deficiency syndrome	Human (8–34 yrs)	3 months classical KD	Increased *Desulfovibrio*, no change in *Firmicutes*, *Bacteroidetes*, *Bifidobacterium*, *Lactobacillus*, *C. perfringens*, *Enterobacteriaceae*, *Clostridium* cluster XIV, or *Faecalibacterium prausnitzii*	Tagliabue et al., 2017^[^ ^155^ ^]^
Epilepsy: Dravet syndrome	Mice	A1783V‐Scn1a mice, 6 weeks KD versus control diet	Increased *Clostridium*, *Acetatifactor*, *Oscillospira*, decreased *Romboutsia* in feces	Miljanovic and Potschka, 2021^[^ ^205^ ^]^
Epilepsy: drug‐resistant epilepsy	Mice	14 days KD (6:1)	Decreased a‐diversity, increased *A. muciniphila* and *Parabacteroides*	Olson et al., 2018^[^ ^12^ ^]^
Epilepsy: infantile spasms syndrome	Rats (neonatal rats)	7 days KD (4:1)	Increased α‐diversity, *Streptococcus*, *Lactococcus*, *Streptococcus infantis*, *Streptococcus lactarius*, *S. thermophilus*, *L. lactis*; decreased *L. johnsonii*, *Escherichia coli*	Mu et al., 2022^[^ ^13^ ^]^
Epilepsy: temporal lobe epilepsy	Rats	Pilocarpine‐induced status epilepticus, 3 weeks KD	Decreased a‐diversity and *Bacteroidetes*, increased *Actinobacteriota*, *Verrucomicrobiota*, and *Proteobacteria*	Li et al., 2024^[^ ^206^ ^]^
Epilepsy	Mice	Pentylenetetrazol‐induced acute seizure model, 8 weeks KD	Decreased short‐chain fatty acids	Eor et al., 2021^[^ ^194^ ^]^
Autistic spectrum disorders	Human (children)	3 months MCT KD	Increased gut‐microbe‐derived trimethylamine *N*‐oxide	Mu et al., 2019^[^ ^6^ ^]^
Autistic spectrum disorders	Human (children)	4 months MCT KD	Increased a‐diversity and *Lactobacillales*, and decreased *Bacteroidaceae*, *Oscillospiraceae*, *Ruminococcus*, *Bacteroides*, *Ruminococcus gnavus*	Allan et al., 2024^[^ ^207^ ^]^
Autistic spectrum disorders	Human	KD (1.95–2.30) (case study, *n* = 1)	Decreased *Firmicutes*, *Bacteroidetes*, and *Proteobacteria*	Bertuccioli et al., 2022^[^ ^208^ ^]^
Autistic spectrum disorders	BTBR T^+^ Itpr3^tf^/J mouse	5 weeks KD	Increased *Akkermansia* and *Blautia* in feces; ameliorated autistic behaviors, reduced proinflammatory cytokines and oxidative stress in hippocampus	Olivito et al., 2023^[^ ^146^ ^]^
Autistic spectrum disorders	BTBR T^+^ Itpr3^tf^/J mouse	10–12 days KD (3:1)	Increased ratio of *Firmicutes*/*Bacteroidetes*, decreased *Bifidobacterium*, *Lactobacillus*, *Roseburia*, *Methanobrevibacter*, *Bacteroides*/*Prevotella* in both cecum content and feces	Newell et al., 2016^[^ ^144^ ^]^
Autistic spectrum disorders	BTBR T^+^ Itpr3^tf^/J mouse	10–12 days KD (3:1)	*Clostridium leptum* negatively correlated with serum ketones and glutathione but positively correlated with glutamine, while *A. muciniphila* positively correlated with serum lactate and taurine, *Bacteroides*/*Prevotella* spp. negatively correlated with serum butyrate	Klein et al., 2016^[^ ^209^ ^]^
Attention deficit hyperactivity disorder	Rats	4 weeks KD	Increased richness and diversity; increased *Ruminococcus gauvreauii* group, *Bacteroides*, *Bifidobacterium*, and *Blautia*; decreased *Lactobacillus*, *Romboutsia*, *Facklamia*, and *Turicibacter*	Liu et al., 2023^[^ ^210^ ^]^
Cognitive impairment	Human	6 weeks high‐fat modified Mediterranean KD versus low‐fat American Heart Association diet	Increased *A. muciniphila*, decreased GABA and GABA‐producing *Alistipes* sp. CAG:514 in feces	Dilmore et al., 2023^[^ ^147^ ^]^
Cognitive impairment	Human	6 weeks high‐fat modified Mediterranean KD versus low‐fat American Heart Association diet	Increased *Enterobacteriaceae*, *Akkermansia*, *Slackia*, *Christensenellaceae*, and butyrate, decreased *Bifidobacterium* and *Lachnobacterium*, and lactate in feces	Nagpal et al., 2019^[^ ^145^ ^]^
Cognitive impairment	Human	6 weeks high‐fat modified Mediterranean KD versus low‐fat American Heart Association diet	Increased fungal diversity and *Agaricus* species, decreased *Saccharomyces* that positively correlates with the change of *Prevotella* and *Ruminococcus*	Nagpal et al., 2020^[^ ^177^ ^]^
Cognitive impairment	Human	6 weeks modified Mediterranean KD	Increased *A. muciniphila*, *Alistipes indistinctus*, and *Bacteroides salyersiae* that positively associated with serum branched‐chain amino acids	Schweickart et al., 2024^[^ ^211^ ^]^
Cognitive impairment	Rats	8 weeks KD	Increased *Proteobacteria*, particularly *Enterobacteriales*	Park et al., 2020^[^ ^212^ ^]^
Alzheimer's disease	APP/PS1 double‐transgenic mice	8 weeks Mediterranean‐ketogenic diet	Increased *Lactobacillus*, *Blautia*, *Intestinimonas*, *Akkermansia*, *Parasutterella*, and decreased *Dubosiella*; microbial metabolites: increased lactate and decrease butyrate and succinate in feces	Park et al., 2024^[^ ^187^ ^]^
Cognitive impairment	Mice	Hypoxia‐induced Cognitive impairment, 7 days KD (6:1)	Increased *B. wadsworthia*, decreased *Clostridium cocleatum*	Olson et al., 2021^[^ ^104^ ^]^
Hypoglycemia‐induced cognitive dysfunctions	Mice	2 weeks KD	Increased *Proteobacteria*, *Dorea*, *Helicobacter*, *Desulfovibrio*, decreased *Lactobacillus*, *Prevotella*, *Butyricicoccus*, *Clostridium*, *Rikenella*	Li et al., 2024^[^ ^213^ ^]^
Parkinson's disease	Mice	MPTP‐induced mouse model, 8 weeks KD (90.5% kcal fat and <0.5% kcal carbohydrate)	KD exerts neuroprotective role, attenuating dopaminergic neuron loss and motor dysfunction and reversing the MPTP‐induced increase in *Citrobacter*, *Desulfovibrio*, and *Ruminococcus* and inflammatory cytokines in the colon. KD decreases α‐diversity	Jiang et al., 2023^[^ ^156^ ^]^
Parkinson's disease	Mice	MPTP‐induced mouse model, 5 weeks MCT KD (89.91% kcal fat and 0% kcal carbohydrate)	Decreased α‐diversity and *Desulfomicrobium*, increased *Blautia*, *Romboutsia*	Zhang et al., 2023^[^ ^157^ ^]^
Traumatic brain injury	Mice	7 days KD	Decreased *L. reuteri* in feces and its metabolite indole‐3‐acetic acid in the large intestine	Dilimulati et al., 2023^[^ ^171^ ^]^
Stroke	Rats	Middle cerebral artery occlusion‐induced stroke; 2 months KD	Increased *Ruminococcaceae*, decreased *Prevotellaceae*, *Bacteroidaceae*, *Acidaminococcaceae*, *Rikenellaceae*, and *Sutterellaceae*; KD slightly worsened the neurological functions in stroke	Zharikova et al., 2024^[^ ^214^ ^]^
Obesity	Human	12 weeks KD versus baseline	Decreased *Eubacterium hallii* group, *Pseudomonas*, and *Blautia*	Yuan et al., 2022^[^ ^215^ ^]^
Obesity	Human	2 months VLCKD	Increased microbial diversity, *Oscillospira*, and *Butyricimonas*, decreased *Enterobacteriaceae*, *Erwinia*, and *Citrobacter*	Gutierrez‐Repiso et al., 2019^[^ ^192^ ^]^
Obesity	Human	6 weeks, randomized, crossover trial Atkins diet	Increased *Actinobacteria*, decreased *Proteobacteria*	Haji‐Ghazi Tehrani et al., 2022^[^ ^216^ ^]^
Obesity with altered intestinal permeability	Human	8 weeks KD	Decreased *Bifidobacterium*, *Ruminococcus*, *Agathobacter*	Celano et al., 2024^[^ ^217^ ^]^
T2DM with overweight or obesity	Human	3 months VLCKD	Increased *Akkermansia*, *Christensenellaceae* family, *Eubacterium* spp., decreased *Firmicutes*, Actinobacteriota, and *Alistipes*	Deledda et al., 2022^[^ ^148^ ^]^
Obesity	Human	1 months KD	Increased *Ruminococcus bromii*, decreased LPS‐producing bacteria	Guevara‐Cruz et al., 2024^[^ ^218^ ^]^
Obesity	Mice	4 weeks KD versus HFD	Increased *Lactobacillus* and *Bacilli*, no impact on SCFA	Dong et al., 2023^[^ ^219^ ^]^
Obesity	Mice	4 weeks KD	Increased *Bacteroides*, *Fusobacterium*, *Escherichia*/*Shigella*, *Intestinimonas*, decreased *Actinobacteria*, *Firmicutes*, *Dialister*, *Streptococcus*, and *Bifidobacterium* particularly *Bifidobacterium adolescentis* with no impact on the production of SCFA in feces	Ang et al., 2020^[^ ^103^ ^]^
Pancreatic cancer	Mice	2 months KD+ gemcitabine versus control	Extended survival, increased *Faecalibaculum* but decreased *Romboutsia* and *Lactobacillus* in feces	Cortez et al., 2022^[^ ^220^ ^]^
Colorectal cancer	Mice	4 weeks KD	Increased *Lachnospiraceae* and *Ruminococcaceae*, and microbe‐derived stearic acid	Tsenkova et al., 2025^[^ ^168^ ^]^
Lung cancer	Mice	Fish oil‐rich KD for 8 weeks (nicotine‐derived nitrosamine ketone‐induced lung cancer)	Reduced lung nodules, improved lung cancer, and increased *Akkermansia*	Elisia et al., 2024^[^ ^221^ ^]^
Melanoma	Mice	12 days KD (4:1)	KD could enhance the efficacy of immune checkpoint inhibitors, increase the abundance of *Eisenbergiella massiliensis*, *A. muciniphila*, *Lactobacillus animalis*, and *Desulfitobacterium*, and decrease *Lactobacillus intestinalis*, *L. reuteri*, and other species within *Lactobacillaceae* family in feces	Ferrere et al., 2021^[^ ^149^ ^]^
Knee osteoarthritis	Mice	8 weeks KD	KD worsen knee osteoarthritis; increased *Akkermansia* and decreased *Lactobacillus*	Dyson et al., 2025^[^ ^222^ ^]^
Hypoglycemia	Mice	14 days KD	Increased *Firmicutes* and *Proteobacteria*, decreased α‐diversity and *Bacteroidetes*	Li et al., 2023^[^ ^141^ ^]^
Polycystic ovarian syndrome	Rat	8 weeks KD	Increased *Proteobacteria* and *Bacteroides*, decreased *Firmicutes*, *Ruminococcus*, *Roseburia*, testosterone, and 7α‐hydroxytestosterone in feces	Wang et al., 2023^[^ ^142^ ^]^
Colitis	Mice	16 weeks KD (89% kcal fat, <1% kcal carbohydrate), 3% DSS‐induced colitis	Increased *Akkermansia*, decreased *Escherichia*/*Shigella* in the colon	Kong et al., 2021^[^ ^94^ ^]^
Herpes simplex encephalitis	Mice	Herpes simplex virus type 1 (HSV‐1) infection, 1 weeks KD (6:1)	Depletion of gut microbes by antibiotics weakens the protection of KD against virus infection	Shan et al., 2023^[^ ^114^ ^]^

The KD is a metabolic therapy widely used to treat epilepsy, particularly forms of epilepsy that do not respond to antiseizure medications. However, the effects of the KD on the gut microbiome, as measured in fecal samples, have shown inconsistent trends. For example, the KD has been reported to reduce the abundance of *Akkermansia* in children with drug‐resistant epilepsy.^[^ ^140^ ^]^ Remarkably, an increase in *Akkermansia* following KD has also been observed in other conditions (Table 1), including autism spectrum disorders,^[^ ^146^ ^]^ cognitive impairment,^[^ ^145^, ^147^ ^]^ type 2 diabetes mellitus,^[^ ^148^ ^]^ melanoma,^[^ ^149^ ^]^ and colitis.^[^ ^94^ ^]^ As a key mucin‐degrading bacterium in the gut,^[^ ^150^ ^]^ *Akkermansia* species such as *Akkermansia muciniphila* is considered a next‐generation probiotic due to its benefits in regulating intestinal inflammation, maintaining barrier function, and promoting metabolic health.^[^ ^151^ ^]^ Since the KD increases this species in most conditions, it is of interest to investigate whether *Akkermansia* has a role in the disease progress.

Sulfate‐reducing bacteria (SRB) represent another group worthy of attention. These bacteria, including *Desulfovibrio*, *Bilophila*, and *Desulfomicrobium*, utilize sulfate or sulfite as electron acceptors to produce sulfide.^[^ ^152^, ^153^, ^154^ ^]^ The KD has been shown to increase SRB in epileptic conditions such as glucose transporter type 1 deficiency syndrome^[^ ^155^ ^]^ and cognitive impairment^[^ ^104^ ^]^ but decreases SRB in mouse models of Parkinson's disease^[^ ^156^, ^157^ ^]^ (Table 1). Notably, expansion of SRB has been mechanistically linked to diseases such as colitis,^[^ ^158^ ^]^ highlighting the need for further research into their role following KD treatment.

Alterations in the microbial functional features have also been found in terms of microbial metabolism and the synthesis of antimicrobial compounds. For example, SCFA, indicators of microbial activity during carbohydrate fermentation, are generally reduced by the KD in patients with epilepsy^[^ ^35^ ^]^ (Table 1). Interestingly, a responder‐based analysis found that patients with good outcomes had higher levels of the butyrate,^[^ ^140^ ^]^ emphasizing the importance of individualized responses and paving the way for patient‐specific precision treatments.

### 4.3 Mechanistic Insights into the Role of Gut Microbiome following the KD

Uncovering the mechanistic role of the gut microbiome in disease pathogenesis remains a significant scientific challenge. However, advancements over the past couple decades have greatly deepened our understanding of the complex mechanisms by which the gut microbiome finely regulates health and disease. In this section, we focus on experimental evidence showing the microbiome mechanistically regulates metabolic or neurological disorders during KD therapy.

#### 4.3.1 Epilepsy

Recent studies have shown that the gut microbiome is involved in the anticonvulsant effects of the KD (Figure 4). Employing an adult mouse model of refractory epilepsy, Olson et al. demonstrated that an increase in *Akkermansia muciniphila* and *Parabacteroides* species after KD administration exerts anticonvulsant effect by decreasing circulating γ‐glutamyl amino acid and increasing the gamma‐aminobutyric acid:glutamate ratio in the hippocampus.^[^ ^12^ ^]^ More recent evidence indicates that the KD alters the gut microbiome by elevating the microbial function of the methyl citrate cycle and l‐proline biosynthesis that can be transplanted to recipient mice and confer protection against seizures.^[^ ^159^ ^]^ The protection provided by the gut microbiome clearly indicates a mechanistic role in epilepsy treatment.

It should be noted that the etiology and pathogenesis of epilepsy is complex, varying across epilepsy types in infancy^[^ ^160^ ^]^ and adulthood.^[^ ^161^ ^]^ Effects of the KD may vary across epilepsy disorders. This is exemplified by distinct microbiome responses in infantile epilepsy. Infantile epileptic spasms syndrome (IESS), also known as West Syndrome, is a developmental epileptic encephalopathy with poor neurodevelopmental outcomes that occurs in the first year of life.^[^ ^162^, ^163^ ^]^ In a neonatal rat model of IESS, a KD formula effectively reduced the burden of seizures by altering the gut microbiota and downregulating indoleamine 2,3‐dioxygenase 1, as well as increasing kynurenic acid that has antiseizure activity.^[^ ^13^ ^]^ The microbes induced by the KD, including *Streptococcus thermophilus* and *Lactococcus lactis*, conferred antiseizure potential, even when administered along with a normal diet.^[^ ^164^ ^]^ Interestingly, microbiota manipulation by the KD and antibiotics was found to stimulate mitochondrial bioenergetics by increasing mitochondrial electron transport chain function in these rats,^[^ ^165^ ^]^ indicating remote regulation of brain function by the gut microbiome. The effects were further validated when the diet was changed. Upon switch to a KD to a normal, chow based diet, the relative abundance of these bacteria was reduced and hippocampal mitochondria bioenergetic improvements were lost.^[^ ^166^ ^]^ This observation suggests the diet switch affects gut microbiome and the efficacy against epilepsy.

#### 4.3.2 Colorectal Cancer

Colorectal cancer is one of the most prevalent causes of cancer‐related morbidity and mortality worldwide, responsible for ≈7% of new cancer diagnoses and 11% of cancer deaths.^[^ ^167^ ^]^ The KD has recently been shown to reduce the burden of colorectal cancer, and a plausible mechanism likely involves the gut microbiome. Specifically, in a mouse model of colorectal cancer, it promotes gut microbes (i.e., *Lachnospiraceae* and *Ruminococcaceae*) that produce stearate, a microbial metabolite capable of inducing apoptosis in cancer cells and decreasing colonic Th17 immune cell populations.^[^ ^168^ ^]^ This finding further indicates the underappreciated role of microbe‐derived lipid in regulating gut health via host–microbe interactions.^[^ ^169^ ^]^ In combination with previous evidence that the KD can influence the severity of colorectal cancer through BHB,^[^ ^120^ ^]^ it seems that both microbiome‐dependent and microbiome‐independent pathways may play a role in the progression of the disease.

#### 4.3.3 Traumatic Brain Injury

Traumatic brain injury (TBI) is caused by a sudden external physical impact to the head or body, significantly impairing proper brain function. The KD has been found to protect against TBI in both human^[^ ^10^ ^]^ and animal models.^[^ ^170^ ^]^ In adolescent rats with mild TBI induced by a lateral head impact, KD administration either pre‐ or postinduction exerted favorable behavioral effects, notably increasing locomotor exploration as well as reducing anxiety‐ and depressive‐like behaviors.^[^ ^170^ ^]^ In a mouse model of repetitive mild TBI induced by controlled, 7 days cortical impact injury, the injury disturbed motor balance, increased *Lactobacillus reuteri* in feces and its metabolite, indole‐3‐acetic acid (IAA), in the large intestine, serum, and brain.^[^ ^171^ ^]^ The injury also led to the activation of the aryl hydrocarbon receptor and Toll‐like receptor 4/MyD88 signaling pathways.^[^ ^171^ ^]^ Intriguingly, these behavioral and microbial effects were inhibited or reduced by a KD^[^ ^171^ ^]^ (Figure 4), highlighting a microbiome–gut–brain connection mediated by tryptophan‐derived IAA.

#### 4.3.4 Multiple Sclerosis

Multiple sclerosis (MS) is an autoimmune disorder of the central nervous system characterized by aberrant breakdown of the protective myelin sheath surrounding nerve fibers and CNS inflammation. Experimental autoimmune encephalomyelitis is a commonly employed animal model of MS. The KD has been found effective in improving the motor disability and reducing brain inflammation in a mouse model of experimental autoimmune encephalomyelitis.^[^ ^172^ ^]^ A recent study demonstrated that the KD and BHB supplementation improved experimental autoimmune encephalomyelitis in a mouse model of MS. This improvement was associated with the enrichment of *Lactobacillus murinus*, a species capable of inhibiting Th17 cell activity through the production of the tryptophan metabolite indole lactate.^[^ ^173^ ^]^ These findings lay a good foundation for future studies to test the potential regulation of specific microbes or metabolites in MS clinical trials.

#### 4.3.5 Obesity

The pathogenesis of obesity involves increased energy harvest from the diet, and recent research highlights the role of the gut microbiome in this process. Lipid absorption in the gut depends on intestinal bile acids, which are modified by microbial enzymes. Bile salt hydrolase (BSH) is a key microbial enzyme that catalyzes the hydrolysis of amide bonds in conjugated bile acids. This process increases levels of nonconjugated bile acids, which can enhance the esterification rate of dietary lipids and thereby accelerate energy extraction. In a mouse model, *L. murinus* ASF361 was found to produce BSH, leading to increased levels of nonconjugated bile acids. KD treatment reduced the abundance of *L. murinus* ASF361 and increased serum conjugated bile acids, including taurodeoxycholic acid and tauroursodeoxycholic acid. These conjugated bile acids were shown to reduce energy absorption by inhibiting the expression of intestinal carbonic anhydrase 1.^[^ ^174^ ^]^ This microbial influence on bile acid metabolism underscores a previously underappreciated pathway through which the gut microbiome regulates lipid absorption and contributes to the pathogenesis of obesity.

While multiple pathways—including neural, metabolic, and immunological—have been implicated in the microbiome–gut–brain axis, the studies discussed above underscore the fact that much of the observed impact is primarily attributed to specific microbes and their metabolites following KD administration. This is likely due to the nature of the KD as a metabolic intervention that alters metabolism both within the gut microbiome and throughout the entire body. Neural and immunological regulation by the gut microbiome may also play a role, and these mechanisms will likely be uncovered as our understanding of the microbiome continues to expand.

### 4.4 Interkingdom Impact of the Ketogenic Diet

The gut microbiome is comprised of a diverse array of interkingdom microorganisms, including bacteria, fungi, protozoa, and viruses. These members interact to maintain gut homeostasis. Among these complex communities, the fungal component, collectively referred to as the mycobiome, has emerged as an important player in gut health, second only to the bacterial microbiome. Recent studies highlight the mycobiome's critical role in immune modulation^[^ ^175^ ^]^ and metabolic health.^[^ ^176^ ^]^ Interestingly, the KD and ketones have been shown to influence the mycobiome significantly.

One notable connection has been observed in cognitive impairment. Patients with mild cognitive impairment exhibit a distinct mycobiome profile, characterized by increased levels of *Sclerotiniaceae*, *Phaffomycetaceae*, *Trichocomaceae*, and *Botrytis*, alongside decreased levels of *Meyerozyma*.^[^ ^177^ ^]^ Remarkably, a modified Mediterranean KD has been reported to modulate the mycobiome, enhancing fungal diversity and increasing the abundance of *Agaricus* species while reducing *Saccharomyces*. These changes positively correlate with shifts in bacterial genera such as *Prevotella* and *Ruminococcus*.^[^ ^177^ ^]^ With the known alterations of fungal members, it will be highly relevant to further investigate how the KD affects the fungal metabolism and immunological functions that might be involved in cognitive health.

Ketone esters, developed to treat epilepsy, offer improved tolerability and rapid ketone delivery to the systemic circulation. In spontaneously epileptic *Kcna1*‐null (KO) mice, which lack the potassium channel α subunit Kv1.1, subcutaneous administration of ketone esters effectively reduced the seizure burden.^[^ ^3^ ^]^ Beyond their neurological effects, ketone esters were found to modulate the gut microbiome by reducing *Bacteroides*, *Lactobacillus*, and *Lachnospiraceae*, while also altering the mycobiome by increasing *Ascomycota* and *Saccharomyces*.^[^ ^3^ ^]^ The implications of these changes remain unclear; however, members of *Saccharomyces*, such as *Saccharomyces cerevisiae* ^[^ ^178^ ^]^ and *Saccharomyces boulardii*,^[^ ^179^ ^]^ have been employed as probiotics to improve conditions like ulcerative colitis and liver disease, respectively. The detailed mechanisms of the mycobiome in metabolic and neurological disorders remain to be elucidated, but warrants further investigation given these observations. Furthermore, although the mycobiome is present in relatively low abundance, it plays an essential role in regulating intestinal immunity and metabolism.

## 5 Therapeutic Implications

While the KD can confer various metabolic and immunological benefits in disease, it can also produce side effects that need to be carefully monitored during administration, such as those in gastrointestinal (e.g., constipation, gut microbiota dysbiosis) and peripheral system (e.g., dyslipidemia, kidney stone, micronutrient deficiencies, alterations in bone metabolism, loss of lean muscle mass, protein‐losing enteropathy). Refinement of the KD by improving tolerability with adjustments to composition and implementation is one avenue to mitigate untoward side effects.

### 5.1 Refining the Ketogenic Diet to Manage Gastrointestinal Side Effects

The route and type of KD administration influence its efficacy and tolerability. Dietary intolerance remains a major barrier to KD adherence in metabolic therapy. To address this, alternative administration methods have been developed. For instance, ketone esters have shown efficacy in controlling seizures while maintaining tolerability in rodent models.^[^ ^180^ ^]^ Recently, we demonstrated that subcutaneous administration of ketone esters not only improved seizures but also influenced the gut microbiome and mycobiome.^[^ ^3^ ^]^ Furthermore, a cyclic KD (administered every other week) reduced midlife mortality and improved memory behaviors in mouse models.^[^ ^181^ ^]^ These findings highlight the potential of refining KD patterns and types to enhance its therapeutic application.

Constipation is another frequently reported side effect following KD therapy, affecting up to 65% (31/48)^[^ ^182^ ^]^ or 69% (18/26)^[^ ^183^ ^]^ of epilepsy patients following KD initiation. Other gastrointestinal side effects, such as nausea, vomiting, and diarrhea, were also commonly observed.^[^ ^182^, ^184^, ^185^, ^186^ ^]^ These conditions necessitate careful monitoring during KD therapy.

Accumulating evidence underscores the decrease of SCFA producers, such as *Roseburia*,^[^ ^144^ ^]^ *Faecalibacterium*,^[^ ^137^ ^]^ and *Bifidobacterium*,^[^ ^140^, ^143^ ^]^ as well as the decreased concentration of SCFA after KD treatment.^[^ ^35^, ^187^ ^]^ Prebiotic or synbiotic^[^ ^188^ ^]^ (mixture of probiotics and prebiotics) administration stimulates the growth of SCFA producers and may offer a solution to restore microbial balance and improve gut health. Encouragingly, dietary fiber or specific prebiotics can regulate gut function and can be integrated with the KD to mitigate these issues while maintaining the therapeutic efficacy of the diet.^[^ ^189^, ^190^ ^]^ A recent study further shows the expansion of potentially pathogenic species after the KD, such as *Clostridium perfringens* that dysregulates bile acid metabolism.^[^ ^191^ ^]^ Addressing these gastrointestinal challenges is essential for optimizing KD adherence and outcomes in clinical practice.

### 5.2 Beyond the Gut: Peripheral Side Effects

Low‐grade systemic inflammation is known as a key etiology in obesity. Synbiotic inclusion has been tested in the context of the KD for regulating peripheral infllamation. In a clinical trial, supplementation of a synbiotic (*Bifidobacterium animalis* subsp. *lactis* plus prebiotic fiber) with the KD reduced inflammation, as reflected by decreases in C‐reactive protein and LPS‐binding protein.^[^ ^192^ ^]^ The benefits conferred by synbiotics may hold promise in reducing KD‐induced side effects.

Dyslipidemia is a common side effect during KD treatment for epilepsy.^[^ ^127^, ^193^ ^]^ This effect is more worrisome in pediatric conditions (e.g., infantile spasms) due to the young age of affected individuals. KD exposure in early life might cause dyslipidemia due to immature metabolism and immunity. For instance, a 3 days ketogenic diet formula induced hepatic steatosis and oxidative stress in neonatal rats with infantile spasms. However, coadministration of a mixture of *S. thermophilus* and *L. lactis* ameliorated these effects by upregulating lipid oxidation and AMP‐activated protein kinase signaling^[^ ^193^ ^]^ (Figure 4). Considering the possible detrimental effects of the KD in infants, prebiotic supplementation has also been tested. Combination of oligofructose‐enriched inulin with KD could maintain the anticonvulsant effects of KD while increasing *Bifidobacterium pseudolongum* and *Lactobacillus johnsonii* in the feces relative to the KD alone.^[^ ^189^ ^]^ Thus, incorporation of targeted probiotics or prebiotics seems to be beneficial to counteract some of the side effects resulting from early life KD treatment. In a pentylenetetrazol‐induced acute seizure model, inclusion of a synbiotic (*Lactobacillus fermentum* MSK 408 with galactooligosaccharide) with the KD reduced serum triglycerides relative to KD alone,^[^ ^194^ ^]^ indicating its potential in lowering lipidemia risk. Overall, developing alternatives based on a stronger understanding of the mechanistic underpinnings of KD action may yield significant advances in the growing field of metabolism‐based treatments for neurological and other disorders.

In addition to dyslipidemia and gastrointestinal distress, other KD‐related side effects warrant attention, such as nephrolithiasis, micronutrient deficiencies, bone demineralization, and “brain fog.”^[^ ^41^, ^195^, ^196^ ^]^ For instance, “brain fog,” namely difficulty in concentration, has been reported in 10.9% of healthy adults after the KD.^[^ ^196^ ^]^ KD may also affect the muscle mass, but the direction of change and potential side effects remain controversial.^[^ ^197^ ^]^ Another rare complication is protein‐losing enteropathy, a condition where there is excessive loss of blood proteins via the gastrointestinal tract.^[^ ^198^, ^199^ ^]^ A clinical case of protein‐losing enteropathy, present as lymphatic ectasia in the intestinal lamina propria, has been reported after a 4:1 KD treatment, with the symptom amelioration after decreasing the ratio to 1.05:1.^[^ ^199^ ^]^ An additional risk involves the cardiac axis, as a MCT‐based KD was reported to cause cardiac fibrosis in a mouse model.^[^ ^200^ ^]^ Unfortunately, current strategies to counteract these complications remain limited. With growing insights into the gut microbiome and metabolism, future research in these areas is essential to enhance the benefits of the KD while minimizing its risks.

It is important to note that the KD has been traditionally employed as a therapy for medically intractable epilepsy and potentially for other neurologic disorders under strict guidance from clinicians in both pediatric populations and adults.^[^ ^201^, ^202^, ^203^ ^]^ Given the potential adverse effects, one should consult their healthcare provider before initiating KD treatment.

## 6 Perspectives and Conclusions

The KD is a well‐established metabolic therapy with broad implications for gastrointestinal physiology and host–microbiome interactions. The diet impacts the digestion and absorption of macronutrients and micronutrients, and its effects on body weight regulation are linked to satiety hormones and the enteroendocrine system. Additionally, the KD may influence gut motility via the serotonin and cannabinoid systems, with potential relevance to epilepsy and IBS. These fundamentally impact the gut and are tightly linked to clinical phenotypes caused by the KD, such as dyslipidemia, mineral deficiency, and constipation. However, while the potential negative impacts are beginning to be uncovered, appropriate interventions to manage untoward consequences remain limited. Future investigations are necessary to understand the mechanisms of action and translate findings from bench to bedside.

Intestinal permeability and barrier integrity are central to gut health, and the KD can exert both positive and negative effects on the severity of intestinal inflammatory diseases, such as IBS and colitis. The effects depend on multiple factors, including KD composition, duration of treatment, animal models used, and different physiological conditions. Available evidence also supports the KD's role in modulating intestinal immunity, including regulation through innate lymphoid cells, T cell subsets, IgA responses, and antiviral immunity. Although few studies are available, the potential applicability of the KD for viral infections opens a new avenue for immunometabolism‐based interventions.

The KD has also been evaluated in preclinical cancer models involving the gastrointestinal tract, including gastric, colorectal, and pancreatic cancers. While these studies suggest potential benefits, more evidence is needed to translate these findings into effective cancer management strategies for humans.

Emerging research highlights KD's impact on intestinal epithelial processes, such as mitochondrial function, intestinal stem cell fate—particularly through ketone bodies and circadian rhythms. Proper mitochondrial and stem cell function in the intestinal epithelium is critical to maintaining gut homeostasis and involves extensive interactions with the gut microbiome and surrounding cells. A deeper understanding of these interactions is invaluable toward elucidating the cellular mechanisms underlying the KD's effects on the gut and microbiome, as well as the mycobiome. Future efforts using intestinal organoids may provide more physiologically relevant and mechanistic insights into these linkages.

In terms of the impact on the gut microbiome, the KD induces location‐specific alterations within the gut and context‐specific effects in various disorders, including epilepsy, autism, colitis, cognitive decline, obesity, and cancer. Alterations in specific microbes may serve as biomarkers of KD responsiveness. Experimental evidence supports a mechanistic role of the gut microbiome in mediating the KD's effects on metabolic disorders (e.g., obesity) and neurodevelopmental disorders (e.g., epilepsy), often through microbiome‐derived molecules such as tryptophan metabolites and bile acids. The effects of the KD on the microbiome have spurred scientific interest in developing microbiome‐based interventions as alternative or complementary treatments. While the microbiome–gut–brain axis has been widely discussed in neurological disorders (e.g., epilepsy), additional experimental proof is still needed. Emerging evidence also supports the interkingdom impact of the KD, particularly the fungal community. Since the gut microbiota interacts closely with fungi and phage populations, it will be worthwhile to understand the role of those underrepresented members.

Given that the KD may induce gastrointestinal and systemic side effects that may affect adherence, identifying and mitigating these adverse effects is an urgent clinical need. Tailoring KD to reduce side effects may involve optimizing administration and composition of the KD and its variants, as well as incorporating microbiome‐targeted approaches such as probiotics, prebiotics, and synbiotic formulations. In summary, these findings posit gut‐mediated mechanisms as a fundamental interface between the KD and systemic health outcomes. Strategies that preserve gut health while enhancing the therapeutic benefits of KD could provide clinically relevant and improved treatments for optimizing disease and health outcomes.

## Conflict of Interest

The authors declare no conflict of interest.

## Author Contributions

C.M. wrote the paper with input from all authors. J.M.R. and J.S. edited the draft. All authors read and approved the final version of this paper.
